# IncobotulinumtoxinA for the treatment of spasticity in children with cerebral palsy – a retrospective case series focusing on dosing and tolerability

**DOI:** 10.1186/s12883-020-01702-7

**Published:** 2020-04-08

**Authors:** Angel León-Valenzuela, Juan Sánchez Palacios, Rogelio del Pino Algarrada

**Affiliations:** 1 Unidad de Gestión Clínica de Rehabilitación, Hospital Puerto Real, Puerto Real, Cádiz, Spain; 2 Grupo iRehab, Instituto de Investigación e Innovación Biomédica de Cádiz, Cádiz, Spain; 3 Departamento Materno-Infantil, Facultad de Medicina, Universidad de Cádiz, Cádiz, Spain; 4 Unidad de Gestión Clínica de Rehabilitación, Hospital Puerta del Mar, Cádiz, Spain

**Keywords:** Spasticity, Cerebral palsy, Pediatric rehabilitation

## Abstract

**Background:**

IncobotulinumtoxinA (Xeomin®) is a botulinum neurotoxin type A with established efficacy in the treatment of upper-limb spasticity in adults. This retrospective case series in a university hospital setting aimed to elucidate the safety and tolerability of incobotulinumtoxinA for treatment of spasticity in children with cerebral palsy.

**Methods:**

Participants received incobotulinumtoxinA injections up to a maximum total dose of 600 U, 24 U/kg body weight. Medical records were reviewed for key demographic information, incobotulinumtoxinA exposure, and adverse effects (AEs).

**Results:**

Sixty-nine children were included (mean age [SD], 8.3 [3.9] years; 44/69 [63.8%] male). One-hundred-and-ninety-one injections were administered, with mean (SD) of 2.8 (1.5) treatment cycles/participant and dosing interval of 6.0 (1.7) months. The number of muscles injected increased from 2.4 (1.2) at cycle 1 to 4.2 (1.9) at cycle 6. The mean (SD) total incobotulinumtoxinA dose increased from 191.7 (126.2) U, (8.5 [5.4] U/kg body weight) at cycle 1 to 368.0 (170.1) U, (9.9 [5.5] U/kg body weight) at cycle 6. Seventy four adverse effects (37.5% of injections) were reported, the most frequent was injection pain (93.2% of AEs). Only three AEs were considered directly treatment-related by injectors: muscle weakness, generalized weakness, and fever.

**Conclusions:**

Our clinical experience indicates that incobotulinumtoxinA is a well-tolerated treatment option for focal spasticity in children with cerebral palsy.

**Trial registration:**

As the study was observational and retrospective, no EudraCT registration number was requested. The internal code assigned to the study in the administrative resolution was: 1143-N-15.

## Background

Cerebral palsy represents a heterogeneous group of permanent movement and posture disorders attributed to non-progressive disturbances that occur in the developing fetal or infant brain [1]. It is one of the most common causes of physical disability in children, occurring in 2.11/1000 live births worldwide [2]. Affected individuals can experience impaired motor control; cognitive delay; and disturbances in sensation, perception, communication, and behavior [1, 3].

One of the most common manifestations of cerebral palsy is spasticity [4, 5], which has been defined as a ‘disordered sensory-motor control, resulting from an upper motor neuron lesion, presenting as intermittent or sustained involuntary activation of muscles’ [6]. Spasticity alters the previously normal skeletal anatomy [5] and can cause contractures, pain, and bone lesions and fragility [3].

Botulinum neurotoxin (BoNT) type A is an effective and well-tolerated treatment option for focal spasticity in children with cerebral palsy [3, 7–9]. It inhibits acetylcholine release from nerve terminals at the motor endplate, preventing synaptic transmission [10]. This causes a temporary reduction in the muscular activity of injected muscles [10] and reduces the pain associated with muscular hyperactivity [11]. Focal injection means that only the symptomatic muscles are treated, with a duration of paresis of around 2 to 3 months [10], thereby limiting systemic side effects [3]. Reduction of spasticity after BoNT type A injection should open a therapeutic window for other interventions in cerebral palsy, by preventing contracture formation and enhancing both motor ability and functional skills.

IncobotulinumtoxinA (Xeomin®, Merz Pharmaceuticals, Germany) is a BoNT type A formulation that is free from complexing proteins and therefore has a high specific neurologic activity [12, 13]. While incobotulinumtoxinA has established efficacy in the treatment of upper-limb spasticity in adults [14–17], there are limited published efficacy and safety data relating to the use of incobotulinumtoxinA for the treatment of spasticity in children with cerebral palsy. However, a recent, randomized, double-blind, comparator-controlled trial in children with cerebral palsy reported a safety profile for incobotulinumtoxinA similar to that of another BoNT type A formulation (onabotulinumtoxinA) [18].

Here, we describe our experience with incobotulinumtoxinA for the treatment of spasticity of the upper and/or lower limb in children with cerebral palsy, particularly focusing on dosing and tolerability.

## Methods

This retrospective case series included children with a diagnosis of infantile cerebral palsy who received up to six treatment cycles of incobotulinumtoxinA for spasticity of the upper and/or lower limb(s) at the authors’ University Hospitals between April 12, 2013 and April 22, 2017. Participants received incobotulinumtoxinA injections up to a maximum total dose of 600 U, 24 U/kg body weight, frequently performed under sedation or anesthesia. Before each injection patients were informed about possible side effects and their management (eg pain or fever and on the use of analgesic or antipyretic medication). Patients were followed up between 4 and 6 weeks after injection. Participants and their caregivers were actively questioned regarding AEs at each post-injection visit. The recording of data was done by the same injector physician and the decision to repeat the infiltration depends on the physician clinical criteria, being carried out at variable intervals of 4 to 6 months.

The study protocol was approved by a local ethics committee and the directors at each participating site. Medical records were reviewed for participants’ demographic details and baseline disease characteristics, doses of incobotulinumtoxinA injected at each treatment session, the number of injection sites, muscles injected, and intervals between consecutive doses. Records were also reviewed for any AEs occurring following incobotulinumtoxinA treatment and for the need for subsequent health care (both in primary and hospital care records). Participants’ legal guardians signed the written consent for the interventions and to participate in the study. The requirement to obtain informed consent from patients and Committee approval for secondary analysis during the pair review was waived because it was classified as a retrospective study on an anonymous database.

Mobility was recorded using the Gross Motor Functional Classification System (GMFCS): level I, walks without restriction, limitations in more advanced gross motor skills; level II, walks without assistive devices, limitations walking outdoors and in the community; level III, walks with assistive mobility devices, limitations walking outdoors and in the community; level IV, self-mobility with limitations, with children transported or using powered mobility outdoors or in the community; and level V, self-mobility is severely limited, even with the use of assistive technology [19]. The ability to handle objects was recorded using the Manual Ability Classification System (MACS): level I, objects are handled easily and successfully; level II, handles most objects, but with somewhat reduced quality and/or speed of achievement; level III, handles objects with difficulty and will need help to prepare and/or modify activities; level IV, handles a limited selection of easily managed objects in adapted situations; and level V, does not handle objects and has severely limited ability to perform even simple actions [20].

### Statistical analysis

The absolute and relative frequencies of qualitative variables are reported. Unless otherwise indicated, quantitative variables were assessed using descriptive statistics and are reported as mean (SD) [range] or as median [range] when values are not normally distributed. Parametric (paired sample *t*-test) and non-parametric (Wilcoxon signed-rank) tests were performed to analyze dose-progression with increasing treatment cycles, with *p* < 0.05 being considered significant.

## Results

### Participants

A total of 69 children with cerebral palsy were included in this study (Table 1). Participants ranged in age from 1.8 to 17.9 years (mean [SD]: 8.3 [3.9] years). Twenty-seven participants (39.1%) presented hemiplegia: 13 with hemiplegia on the right side, 11 with hemiplegia on the left side, and three with laterality undefined. Of the remaining participants, 26 (37.7%) presented tetraplegia and 16 (23.2%) diplegia. The greatest proportion of participants presented with Gross Motor Functional Classification System (GMFCS) level II (28/68, 41.2%; data missing for 1 participant); however, 25/68 (36.8%) presented with GMFCS level IV or V. Most participants presented with Manual Ability Classification System (MACS) level I or level II (36/64, 56.2%; data missing for 5 participants); however, 18/64 participants (28.2%) presented with level IV or V. No changes were reported in the number of participants in each GMFCS or MACS classification level following repeated incobotulinumtoxinA injections.

**Table 1:** Participant demographics and baseline disease characteristics

Characteristic	Participants (*n* = 69)
Sex, *n* (%)	
Female	25 (36.2)
Male	44 (63.8)
Mean age, years (SD) [range]	8.3 (3.9) [1.8–17.9]
Median body weight, kg [range]	22.0 [10.0–78.0]
Spasticity diagnosis, *n* (%)	
Hemiplegia	27 (39.1)
Tetraplegia	26 (37.7)
Diplegia	16 (23.2)
GMFCS, *n* (%)^a^	
Level I	10 (14.7)
Level II	28 (41.2)
Level III	5 (7.4)
Level IV	10 (14.7)
Level V	15 (22.1)
MACS, *n* (%)^b^	
Level I	21 (32.8)
Level II	15 (23.4)
Level III	10 (15.6)
Level IV	6 (9.4)
Level V	12 (18.8)

^a^Data missing for one participant; ^b^Data missing for five participants. *GMFCS* Gross Motor Function Classification System, *MACS* Manual Ability Classification System, *SD* standard deviation

### Treatment

A total of 191 incobotulinumtoxinA injections were administered, with a mean (SD) of 2.8 (1.5) treatment cycles per participant (Table 2). The mean (SD) treatment duration was 10.6 (9.4) months (range 0–33.6), and the mean (SD) interval between consecutive treatment cycles was 6.0 (1.7) months.

**Table 2:** IncobotulinumtoxinA dosing at each treatment cycle

No. of Treatment cycle/patient	Participants, *n*	No. of muscles injected; mean (SD) [range]	IncobotulinumtoxinA dose injected, U; mean (SD) (range)	IncobotulinumtoxinA dose injected, U/kg;mean (SD) [range]	Maximum total dose recommended^a^, U; mean (SD) [range]	Percentage of maximum total dose injected^b^, %; mean (SD) [range]
1	69	2.4 (1.2) [1–5]	191.7 (126.2) [40–600]	8.5 (5.4) [0.7–24]	323.3 (82.9) [160–400]	59.2 (33.2) [10–150]
2	52	2.6 (1.6) [1–7]^c^	179.2 (114.0) [40–420]	7.6 (4.9) [0.7–17.5]	338.1 (77.5) [176–400]	53.4 (29.6) [10–109]
3	33	3.0 (1.6) [1–7]^d^	225.8 (130.4) [40–480]	8.6 (5.2) [0.7–17.6]	356.1 (67.1) [192–400]	63.2 (31.8) [10–120]
4	21	3.4 (1.7) [1–7]	258.6 (128.6) [80–480]	9.3 (4.8) [3–15.7]	363.5 (65.4) [192–400]	71.5 (31.5) [20.3–120]
5	11	3.6 (1.6) [1–6]	286.4 (134.5) [80–480]	9.7 (4.5) [3–16]	360.0 (81.7) [192–400]	80.4 (31.2) [20–120]
6	5	4.2 (1.9) [1–6]	368.0 (170.1) [80–500]	9.9 (5.5) [3–15.4]	400.0 (0.0) [400–400]	92.0 (42.5) [20–125]

^a^Maximun total dose/weight recommended in previous articles (16 U/kg, not exceeding 400 U); ^b^Total dose injected/maximum total dose; ^c^*n* = 51; ^d^*n* = 32

IncobotulinumtoxinA dosing details are summarized in Table 2. The mean (SD) number of muscles injected increased from 2.4 (1.2) at treatment cycle 1 (*n* = 69) to 4.2 (1.9) at treatment cycle 6 (*n* = 5) (*p* < 0.05 for cycles 3, 4, and 5 compared with cycle 1). Compared with the total dose injected at the first treatment cycle, the total incobotulinumtoxinA dose injected increased significantly with subsequent treatment cycles (aside from cycle 2), from a mean (SD) 191.7 (126.2) U, (8.5 [5.4] U/kg) in treatment cycle 1 to 368.0 (170.1) U, (9.9 [5.5] U/kg) in treatment cycle 6 (*p* < 0.05 for cycles 3 and 5). From treatment cycle 1 to treatment cycle 6, the mean total injected incobotulinumtoxinA dose increased from 59.2 to 92.0% of the maximum dose permitted based on participant body weight (24 U/kg; maximum of 600 U; *p* < 0.05 for all cycles aside from cycle 2).

The most frequently injected muscles across all treatment cycles were gastrocnemius (68.1% of participants), hamstrings (47.8%), adductor longus (42.0%), flexor carpi radialis (18.8%), and adductor magnus (18.8%) (Table 3).

**Table 3:** Distribution of muscles injected across all treatment cycles

Injected muscle, *n* (%)	Injections *n* = 191	Participants *n* = 69
Gastrocnemius	136 (71.2)	47 (68.1)
Hamstrings	74 (38.7)	33 (47.8)
Adductor longus	79 (41.4)	29 (42.0)
Flexor carpi radialis	35 (18.3)	13 (18.8)
Adductor magnus	34 (17.8)	13 (18.8)
Pronator teres	33 (17.3)	9 (13.0)
Biceps	30 (15.7)	9 (13.0)
Gracilis	9 (4.7)	9 (13.0)
Psoas	22 (11.5)	8 (11.6)
Adductor pollicis	13 (6.8)	6 (8.7)
Flexor carpi ulnaris	18 (9.4)	6 (8.7)
Opponens pollicis	10 (5.2)	4 (5.8)
Flexor digitorum superficialis	5 (2.6)	3 (4.3)
Soleus	4 (2.1)	3 (4.3)
Brachioradialis	5 (2.6)	2 (2.9)
Tibialis posterior	4 (2.1)	2 (2.9)
Flexor pollicis longus	4 (2.1)	2 (2.9)
Flexor digitorum profundus	2 (1.0)	1 (1.4)
Pronator quadratus	1 (0.5)	1 (1.4)

The proportion of children requiring sedation for injection increased at each treatment cycle from 37/69 (53.6%) at treatment cycle 1 to 4/5 (80.0%) at treatment cycle 6.

### Safety

Seventy four adverse effects (37.5% of injections) were reported, the most frequent was injection pain (93.2% of AEs). Other three AEs reported were considered directly treatment-related by injectors: one report each of generalized mild weakness that resolved within 2 weeks without treatment, fever of 2 days’ duration managed with antipyretics, and localized muscle weakness at adductor level lasting for 4 weeks, which was controlled by reducing the incobotulinumtoxinA dose at subsequent injections. The remaining two AEs (anxiety crisis and vomiting occurring immediately after the wake from sedation of after treatment cycles 3 and 5, respectively) could be more related to sedation. The need for health care due to AE after infiltration was not found in the electronic health records.

## Discussion

BoNT type A is an established, effective, and well-tolerated component of the multimodal treatment of focal spasticity in children with cerebral palsy [3, 7–9]. Nevertheless, there is no uniform strategy for the use of BoNT type A treatment in cerebral palsy, with wide variability seen in dosages [8]. This multimodal treatment approach requires a rigorous assessment of the individual, including their level of motor function, pain, and potential for further physical development [8].

Several BoNT type A treatments are currently available, including incobotulinumtoxinA, which is free from complexing proteins [12, 13]. Only one published study has evaluated incobotulinumtoxinA in children with cerebral palsy and muscle spasticity [18]. This randomized, double-blind study involved 35 children with spastic hemiplegia or diplegia who received either a single dose of incobotulinumtoxinA or onabotulinumtoxinA, injected into the gastrocnemius muscles. The study aimed to establish the safety profile of incobotulinumtoxinA. All of the reported AEs were considered to be minor with no significant difference in the frequency or type of events between treatment groups. Fatigue was the most frequently reported AE. The rate of adverse events reported in this study is higher than that of previous studies, this could be due to the use of a more specific methodology for the detection of adverse events and the use of a detailed list of the adverse events of special interest, which would facilitate detection and reporting. On the other hand, one of the main adverse effects reflected is the pain associated with the injection, which in our case is partially alleviated by the use of sedation during the procedure and by the instruction on pain management before injection. In opposition to this trial, in a recent Cochrane review, Blumetti [21] analyzed 31 studies that included a total of 1508 participants and concluded that the rate of adverse events with botulinum toxin use is similar to the rate obtained with placebo.

In our retrospective case series, we describe our experience with incobotulinumtoxinA treatment for focal spasticity of the upper and lower limbs in children with cerebral palsy, with a focus on dosing and tolerability. A total of 69 participants received at least one treatment cycle. The mean number of injected muscles increased from the first treatment cycle to subsequent cycles, as did the total dose of incobotulinumtoxinA injected per participant (aside from in cycle 2). This is likely to be a reflection of the increased bodyweight of the children as they grew, resulting in a larger absolute maximum dose of incobotulinumtoxinA, which may allow treatment of a greater number of muscles contributing to a particular clinical pattern. Furthermore, consistent with a parallel increase in the proportion of participants receiving sedation and the number of injected muscles with increasing treatment cycles, treatment of a greater number of muscles may be possible when participants are treated under sedation.

Our experience demonstrates that incobotulinumtoxinA treatment for focal spasticity is well tolerated in children with cerebral palsy, consistent with previous results [18]. The fact that no health care was required (neither primary nor hospital care) is indicative of the absence of serious side effects. All three AEs that were considered to be treatment-related occurred in the first treatment cycle. Previous research has suggested that BoNT treatment is less well tolerated in participants with GMFCS levels IV and V [21–23]. A substantial proportion (36.8%) of participants in our study had GMFCS levels IV and V; however, no safety concerns were noted.

### Study limitations

The main limitations of this study include the retrospective design involving the lack of a placebo control. Another limitation inherent to the study design may be the lack of systematic collection of concomitant drugs or AEs, that have been attempted to compensate for the assessment of health records and the need for post-injection health care. Similarly, we can consider a limitation of the study the lack of standardized evaluation of the response to treatment (for example, with the Modified Ashworth or Tardieu Scales) although this was not one of the main objectives of the study. Even though our results indicate that incobotulinumtoxinA injections were well tolerated in this population, further placebo-controlled studies are required to confirm these findings and to assess efficacy.

## Conclusion

In conclusion, our clinical experience indicates that incobotulinumtoxinA is well tolerated in the treatment of focal spasticity in children with cerebral palsy and demonstrates the variation in target muscles and dosage/dosing frequency seen in clinical practice. The study reflects clinical practice, whereby patients are treated according to their individual clinical needs. More extensive prospective studies are required to confirm the safety and efficacy of incobotulinumtoxinA in this population.

## Data Availability

The datasets generated and analysed during the current study are available in the Mendeley. Repository with the identifier: 10.17632/dzdvm63z43.1

## Abbreviations

AE: Adverse events
BoNT: Botulinum neurotoxin
GMFCS: Gross Motor Function Classification System
MACS: Manual Ability Classification System
SD: Standard deviation
